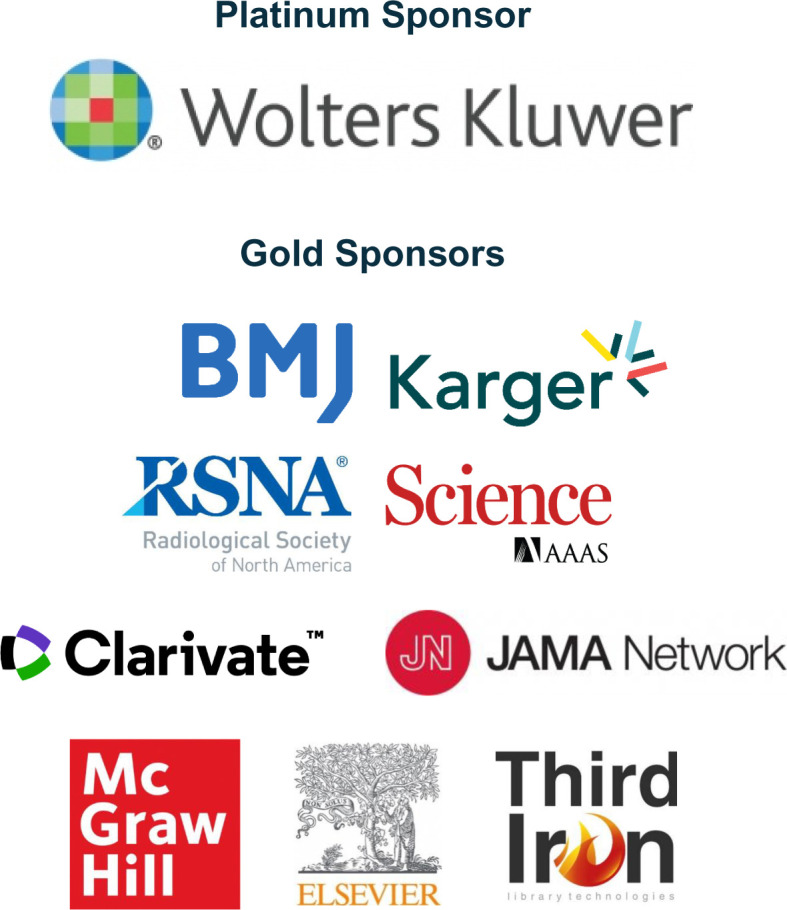# CHLA 2024 Conference Sponsors / ABSC Congrès 2024 Partenaires Industriels

**DOI:** 10.29173/jchla29787

**Published:** 2024-08-01

Thank you to the 2024 conference sponsors!

Your generous and steadfast support for the CHLA/ABSC is appreciated.